# Image Guidance-Assisted Decompression and Removal of Heterotopic Ossification Following the Use of Recombinant Human Bone Morphogenetic Protein-2 in Transforaminal Lumbar Interbody Fusion

**DOI:** 10.7759/cureus.20045

**Published:** 2021-11-30

**Authors:** Julie L Chan, Robert A Ravinsky, J Patrick Johnson, Eli M Baron

**Affiliations:** 1 Department of Neurosurgery, Cedars-Sinai Medical Center, Los Angeles, USA; 2 Department of Orthopaedic Surgery, The CORE Institute, Phoenix, USA

**Keywords:** rhbmp2, bmp, image guidance, navigation, heterotopic ossification

## Abstract

Heterotopic ossification (HO) following the use of recombinant human bone morphogenetic protein-2 (rhBMP-2) in the setting of transforaminal lumbar interbody fusion (TLIF) or posterior lumbar interbody fusion (PLIF) is a troublesome and well-described postoperative complication. There is currently no consensus regarding the treatment of this offending pathology. In this report, we present a retrospective single-surgeon review of 14 patients who underwent image-guided decompression of HO. We describe a new technique where navigation demonstrates a safe and thorough decompression compared to that with fluoroscopy or anatomical landmarks alone. To evaluate successful decompression, we reviewed patient self-reported clinical outcomes. Seven patients demonstrated positive results, while three had mixed outcomes and four showed poor outcomes. While more studies are needed to determine the overall efficacy of intraoperative navigation-assisted decompression, findings from this small cohort of patients suggest that it is a useful technique in the setting of the removal of heterotopic bone.

## Introduction

First discovered and characterized by Urist in the 1970s [1], bone morphogenetic protein (BMP) has been well-studied on a molecular and cellular level, and used clinically as an adjunct to aid in bony unions and arthrodesis in a variety of different settings. Some procedures that have employed BMP include craniofacial surgery, the union of diaphyseal fractures and nonunions, and spinal arthrodesis [2-5].

Since its approval by the US Food and Drug Administration (FDA) for use in anterior lumbar interbody fusions (ALIF), the off-label use of recombinant human bone morphogenetic protein-2 (rhBMP-2) has increased steadily throughout the United States [6,7]. Its use is supported based on the evidence that rhBMP is an effective agent in enhancing rates of fusion throughout the spine with an efficacy comparable to, or even superior to, that of autologous iliac crest bone graft (ICBG) [8]. However, despite enhancing spinal fusions in a variety of clinical scenarios, there are some adverse effects associated with rhBMP in the lumbar spine, such as seroma and/or cyst formation, and inflammatory and/or structural radiculitis, often secondary to ectopic or heterotopic ossification (HO) [9-11].

While there is substantial literature describing HO in the spine and its deleterious sequelae, there is a dearth of literature with respect to guiding the management of this clinical entity. It is postulated that surgical decompression for new-onset radicular symptoms with radiographic evidence of bony structural neurologic encroachment may be indicated; however, revision decompression in this setting can be difficult and may lead to surgical complications. Specifically, there is a high risk of cerebrospinal fluid (CSF) leak and iatrogenic neurologic injury, likely due to scar formation, alteration of the anatomical bony landmarks, and formation of heterotopic bone. Furthermore, the HO may be adherent to the dural sheath and underlying neurologic structures.

In this retrospective study, we present a series of patients who have undergone prior spinal arthrodesis with rhBMP-2 and subsequently developed radiographically-confirmed bony compression of the neurologic elements secondary to HO formation. This study is the first of its kind in the literature to describe the technique that utilizes image guidance for a thorough, safe, and effective decompression that may minimize postoperative complications.

## Technical report

A retrospective review of a single surgeon’s practice (EMB) spanning the period from January 2013 to September 2018 was performed. We identified adult patients with recurrent lumbar spinal stenosis with leg-dominant symptoms who had previously undergone spinal arthrodesis using rhBMP-2. Patients included for analysis demonstrated evidence of recurrent lumbar stenosis due to HO with bony encroachment of the neural elements on CT scan. Patients with recurrent stenosis secondary to progressive degenerative changes, or spondylosis, were excluded from the study. All patients underwent a course of nonoperative treatment of analgesics and steroid injections for a minimum of six months. Those with persistent symptoms of radicular pain greater than 5/10 were considered for revision decompression surgery. Three patients had their index surgeries performed by surgeons other than the senior author. Postoperatively, patients were followed up for a minimum of six months. The Institutional Review Board waiver for study-related consent was obtained.

Data collection

Patient data were collected retrospectively from the electronic medical record and/or the senior author’s patient dossier. Data included demographic information (age at index surgery, sex assigned at birth), type of index surgery, timing and duration of leg symptoms, the timing of revision decompression with respect to both the index surgery and the onset of symptoms, outcome of revision decompression, and complications. Per our Institutional Review Board guidelines, data were collected, analyzed, and stored in a secure electronic database.

Outcomes

Patients were characterized as having a good, poor, or “other” outcome (Table 1). A good outcome was defined as a postoperative reduction of at least 50% of their chief complaint of leg-dominant pain. A poor outcome was defined as persistent leg-dominant pain following surgical decompression. Those that experienced an operative complication and/or complained grossly of a new symptom over a persistent or reduced chief complaint of leg-dominant pain were categorized as “other”.

**Table 1:** Patients who underwent navigated-heterotopic ossification removal following spinal arthrodesis with rhBMP-2 Patients outcomes: 1-7 - good; 8-10 - "other"; 11-14 - poor *Index surgery completed by a surgeon other than the senior author DCS: dorsal column stimulation; DLIF: direct lumbar interbody fusion; HO: heterotopic ossification; LBP: low back pain; LLE: left lower extremity; PSF: posterior spinal fusion; RLE: right lower extremity; TLIF: transforaminal lumbar interbody fusion

	Age in years (index)	Sex	Initial operation	Level/pathology	Clinical presentation	Time to HO removal surgery from initial fusion surgery (months)	Clinical outcome
1	31	M	L4-5, L5-S1 TLIF	L L5-S1 HO	LBP, LLE radiculopathy	48	LLE radiculopathy improved
2	63	M	L2-3 TLIF	L L2-3 HO, L3 HO	LLE radiculopathy	49	LLE radiculopathy improved
3	60	M	L5-S1 TLIF	L L5 HO	LLE radiculopathy, DF 3/5	54	L DF 4+/5, mild improvement in radiculopathy
4	66	M	L4-5 TLIF	L L4-5 HO	LLE radiculopathy	22	LLE radiculopathy improved
5	57	M	L3-L5 DLIF, L5-S1 TLIF	L5-S1 HO	RLE radiculopathy	15	RLE radiculopathy resolved, now with LLE radiculopathy
6	31	F	L4-5 TLIF	L4-5 HO	LBP, RLE radiculopathy	48	RLE radiculopathy resolved
7	54	F	L5-S1 TLIF	L L5-S1 HO	LBP, LLE radiculopathy	23	LBP and radiculopathy improved
8	76	F	L4-5, L5-S1 TLIF	L L5-S1 HO	LLE radiculopathy	42	Radiculopathy improved, then returned
9	59	F	L5-S1 TLIF	L L5-S1 HO, S1 HO	LLE radiculopathy	11	Radiculopathy with minor improvement
10	38	M	L5-S1 TLIF	L L5-S1 HO, L5 HO	LBP, LLE radiculopathy	33	Leg pain improved, back pain stable
11	47	F	L5-S1 PSF	R L5-S1 HO	RLE radiculopathy	45	Bilateral leg pain
12	39	F	L5-S1 TLIF	L L5-S1 HO, S1 HO	Bilateral leg pain	24	Chronic pain, bilateral leg and foot pain, eventually benefited from DCS
13	63	F	T10-L3 PSF*	L L5-S1 HO	Bilateral foot weakness	83	Minimal improvement in weakness
14	48	F	R L4-5 TLIF*	R L4-5 HO	RLE radiculopathy	21	Continued LBP and radiculopathy, possible nonunion, surgery complicated by durotomy

Surgical technique for navigation-augmented decompression

After a standard “time out” typical for our institution, administration of antimicrobial prophylaxis, and the induction of general anesthesia, the patient was positioned prone on a radiolucent Jackson table. The patient was then prepped and draped in the usual sterile fashion. A posterior skin incision was made, taking care to utilize the prior incisional scar. The incision was carried through subcutaneous tissue and adipose, down to the fascia. The fascia was then incised, and careful dissection of the bony elements was employed to reduce the risk of unintentional durotomy or injury to the neural elements. The previous pedicle screw-rod constructs were exposed and the correct level was confirmed by utilizing the existing pedicle screws and/or O-arm navigation.

**Figure 1:**
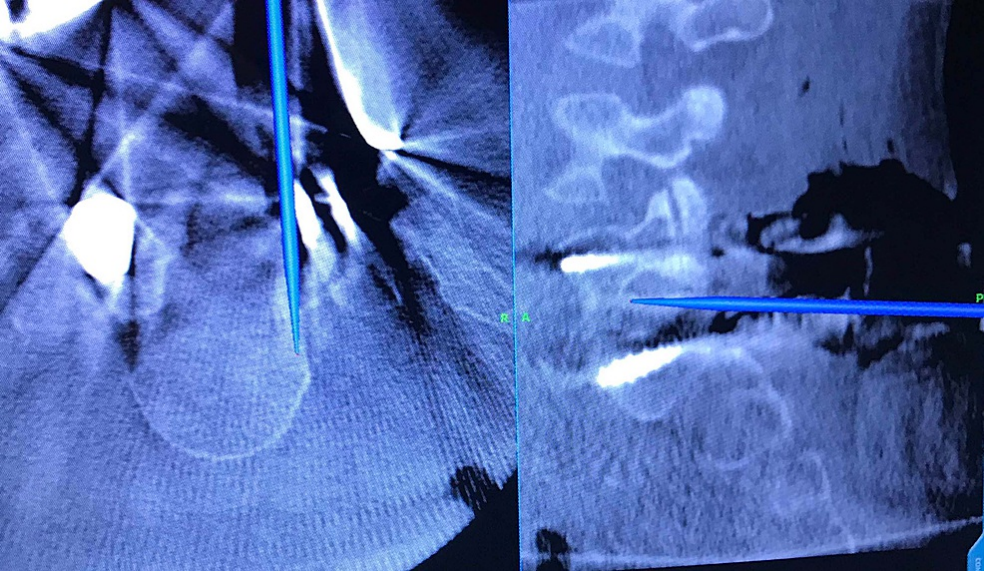
O-arm intraoperative imaging with navigated probe demonstrating HO causing right L5 foraminal stenosis HO: heterotopic ossification

The reference frame for the O-arm navigation system (Stealth, Medtronic, Memphis, TN) was either clamped to a spinous process cranial to the level(s) of interest or secured in the existing tulip heads of the contralateral side. The O-arm was then brought in and a standard-definition image of the levels of interest was performed. Immediately after obtaining the intraoperative CT, a navigated probe was brought into the field to accurately identify the standard bony landmarks as well as the HO elements contributing to neural compression (Figure 1). Using standard microsurgical instruments under the operating microscope, an image-guided revision decompression of the affected area was performed. In a typical case, an extensive dorsal and ventral decompression of the traversing nerve would ensue. A bur would be used to drill a channel in the vertebrae, usually at the level of the disc space. Down-pushing curettes would be used to push the HO bone in the created channel. This often included the use of a mallet on the down-pushing curette while an assistant maintained a root retractor, #4 Penfield dissector, or a Woodson as protection for the neural elements. If necessary, upon completion of the decompression, a second O-arm spin could be performed to confirm the extent of the decompression and ensure that all offending heterotopic bone had been removed. Following decompression, the wound was copiously irrigated and closed in layers. A subfascial drain was placed, sterile dressings applied, and the patient was extubated and taken to recovery. Postoperatively, the patient was followed up closely as an inpatient and outpatient at two, six, 12, and 24 weeks.

Case example

**Figure 2:**
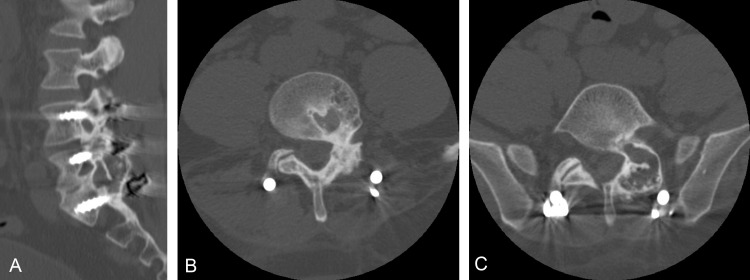
Preoperative sagittal (a) and axial images demonstrating left L4 (b) and left L5 (c) foraminal stenosis

We present the case of a 35-year-old male with a history of minimally invasive transforaminal lumbar interbody fusion (TLIF) via bilateral Wiltse approaches at L4-5 and L5-S1 for back pain who complained of new-onset low back pain, left lower extremity radiculopathy, and left thigh numbness. It was noted that during the initial surgery, 4 mg of rhBMP-2 had been used in each cage. The initial postoperative evaluation had demonstrated improvement in pain and solid fusion on CT. Four years postoperatively, he presented with progressively worsening back pain associated with left thigh numbness and trace left dorsiflexion weakness on exam. Repeat MRI and CT demonstrated significant HO causing severe stenosis of the left L4 and L5 neural foramen (Figure 2). Despite conservative therapy including spinal injections, his pain persisted and so he elected to undergo image-guided decompression of the HO.

**Figure 3:**
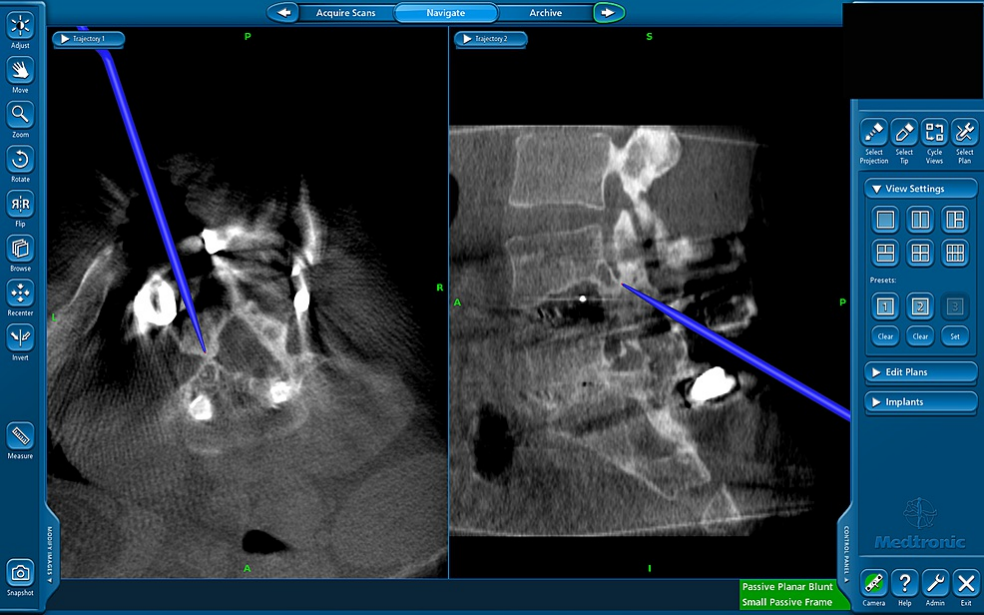
Intraoperative O-arm imaging with navigation probe to assist in the identification of heterotopic ossification

At surgery, his left rod was removed and a spinous process clamp with fiducials was placed at L1 through another incision. A standard definition O-arm image was obtained, and the operating microscope was brought into the room (Figure 3). The navigation probe was used to identify the HO, which was then readily removed with a combination of the drill, down-pushing curettes, and rongeurs. Postoperatively, the patient had marked improvement in his radicular symptoms, which was maintained at his one-year follow-up.

**Figure 4:**
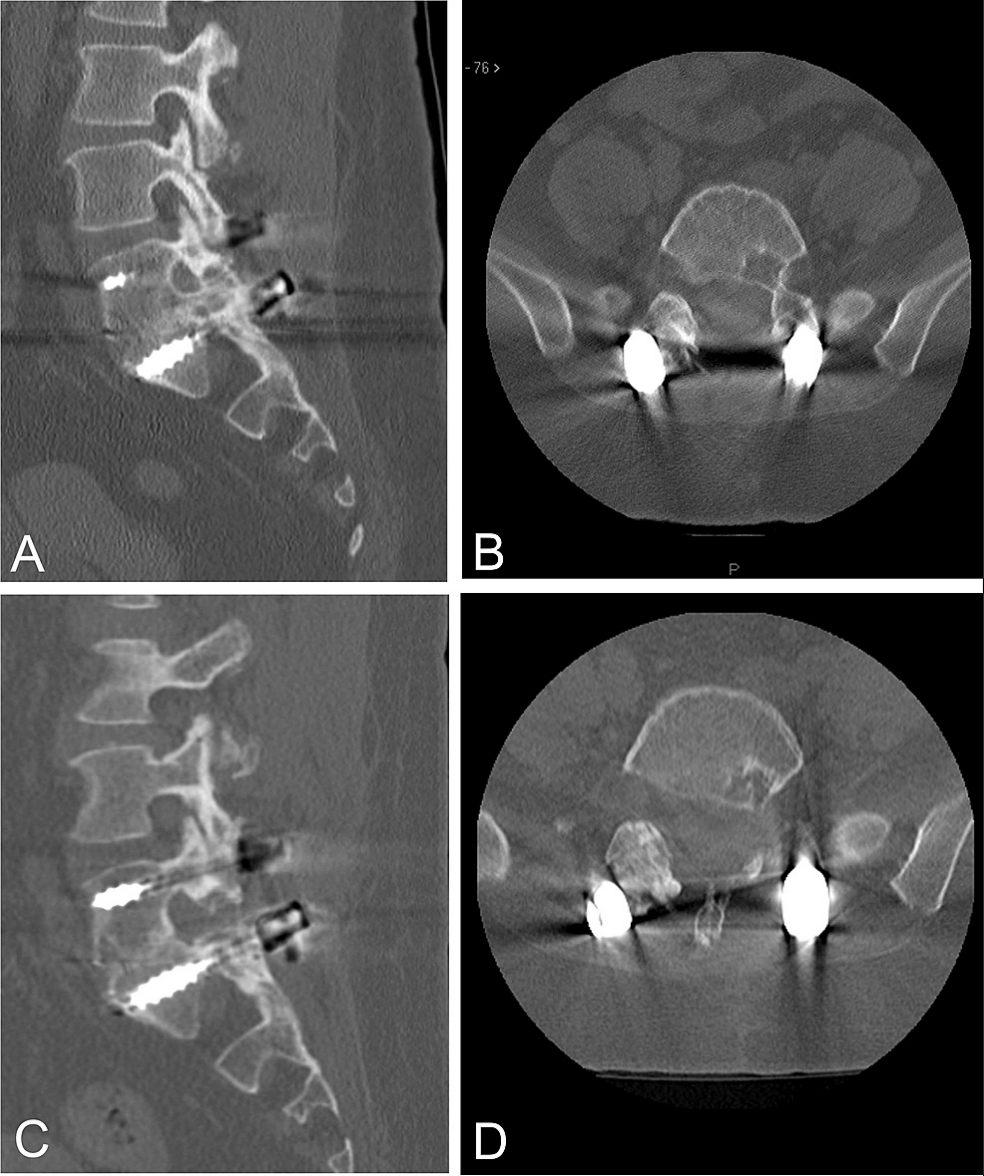
Pre- and postoperative sagittal and axial CT images Preoperative sagittal (a) and axial (b) CT images demonstrating left L5 neuroforaminal stenosis in a patient with recurrent radiculopathy following rhBMP-2 use in spinal fusion. Postoperative sagittal (c) and axial (d) CT images demonstrating appropriate decompression of HO in the left L5 foramen with minimal residual ectopic bone CT: computed tomography; HO: heterotopic ossification; rhBMP-2: recombinant human bone morphogenetic protein-2

We also discuss 14 patients who underwent revision decompression for symptomatic neurologic compression due to HO. All of them underwent navigation-assisted decompression with adequate HO removal as demonstrated in the representative preoperative and postoperative imaging (Figure 4). Seven patients in the cohort were noted to be clinically improved post-revision decompression, while three patients did not show any clinical improvement, and three patients fell into the category of “other”. Of the patients who were determined to have poor outcomes, three had symptoms present for two years or longer. One patient had an unintended durotomy, and one patient had a wound infection.

## Discussion

Following the FDA approval for the use of rhBMP-2 in the LT cage for ALIF [7,12], its utilization has become increasingly common in the promotion of lumbar interbody fusion. The FDA approval was based on studies by Burkus et al. and Boden et al., which specifically evaluated the use of rhBMP-2 in anterior approach spinal arthrodesis as the intervertebral space provided a greater surface area for fusion, and clinically a painful disc could also be removed [13,14]. Since then, the off-label use of rhBMP-2 in TLIF/PLIF and posterolateral fusion has gained traction. However, this has presented new challenges as rhBMP-2 has been associated with radicular complications in both the acute and late perioperative period [15-17]. These adverse events are likely related to rhBMP-2 not only promoting fusion but excessive bony growth and HO. Regarding off-label use in TLIF/PLIF, the reported rates vary significantly from 0-75% and even involve asymptomatic cases [18]. While the factors and exact mechanism by which rhBMP-2 induces HO following TLIF/PLIF in some patients and not others remain to be determined, there does appear to be a dose-dependent effect [19,20].

While the complete prevention of rhBMP-2-induced HO in spinal interbody fusion is ideal, reports suggest that even with the use of precautionary techniques, HO may still occur. To reduce the risk of HO formation, preventive practices may include lower doses of rhBMP-2 to decrease the incidence of cyst formation and possibly HO [19], and irrigation of the surgical field after cage insertion (with rhBMP-2) to reduce inflammation in the spinal canal by washing away any unbound rhBMP-2 [21]. In addition, avoidance of excessive intraoperative blood loss may also decrease the risk of HO as uncontrolled bleeding could result in the migration of rhBMP-2 toward the spinal canal [22]. Investigations into synthetic sealants and barriers regarding their potential as prophylactic measures should aim to determine their safety and efficacy to decrease postoperative radiculopathy related to rhBMP-2. Finally, rhBMP-2 should be placed as far away from the spinal canal as possible [9], although some studies suggest that HO formation is independent of the location of rhBMP-2 placement during spinal arthrodesis [23]. The methods described above may reduce the risk of HO formation after the application of rhBMP-2 in spinal arthrodesis; however, no studies to date have definitively demonstrated the complete prevention of HO. Given the potentially inevitable formation of symptomatic HO, our team focused on methods to reduce the effects of HO during revision surgery.

In the face of symptomatic extraneous bony formation, Ahn et al. described a minimally invasive approach for the resection of HO by using fluoroscopy to skeletonize the iatrogenic bony elements for a complete decompression of the exiting and traversing nerve roots [24]. While fluoroscopy guidance has been demonstrated as useful in the HO removal technique, we found that utilizing O-arm navigation provided a more precise location of the bony elements leading to improved decompression. Specifically, the advantage of increased accuracy of O-arm with image-guidance technology in HO removal may reduce the risk of durotomy by identifying abnormal anatomy in the setting of scarred elements prevalent in revision surgery.

With respect to the technical ease of revision decompression in the setting of HO, our experience showed that the addition of intraoperative CT-based navigation greatly improved the completeness of the decompression and the confidence with which decompression was achieved. In the absence of intraoperative navigation, a surgeon must only rely on preoperative cross-sectional imaging and known intraoperative landmarks to safely decompress the neural elements without surgical misadventure. While nerve root anatomy may vary in the lumbar spine, one cannot overstate the importance of a sound working knowledge of the anatomic relationships between the pedicles, the foramina, and the exiting and traversing nerve roots. This is especially true in patients who have undergone prior surgical decompression, where even in the setting of scar overlying dura, one can reliably locate the pedicle and use this to identify the foramen cranially, and the traversing nerve root medially. The shortcoming of this technique, however, is that in the setting of HO, these normal anatomic relationships may be dramatically altered. Furthermore, with the increasing off-label use of rhBMP-2 and, consequently, increasing frequency of patients presenting with recurrent stenosis due to HO, navigation-augmented decompression can potentially increase the safety of these complex revision decompression procedures.

This retrospective series demonstrated the potentially improved safety of employing intraoperative CT in the decompression of HO caused by rhBMP-2 following TLIF. Out of 14 patients who underwent revision decompression surgery for removal of HO, two had surgical complications: a durotomy and a wound infection. None sustained any new neurologic deficit with the procedure. In terms of outcomes, the decompression appeared effective in more than half of the patients, somewhat effective in two patients, and ineffective in four patients. Of note, 75% of the patients without symptomatic improvement were those who had presented with chronic symptoms for more than two years.

This study has several limitations. While our results suggest that surgical outcomes in HO-related radiculopathy may be marginal at best, our study is likely inadequate to draw any definitive conclusions. Firstly, the data we have presented was collected retrospectively, and hence susceptible to hindsight bias. Secondly, we presented a small, heterogeneous series of patients presenting with similar clinical complaints due to a singular pathologic etiology. While a definitive analysis of the utility or outcomes using this technique will require a larger cohort of patients, our main objective was to describe an alternative safe surgical technique to reduce the radiographic and clinical sequelae following rhBMP-2 use in spinal arthrodesis. It appears likely that the longer duration of symptoms may be a risk factor for poor outcomes regardless of the adequacy of decompression. It is our hypothesis that the clinical prognosis of bony stenosis due to rhBMP-2 may even be more deleterious than other causes of lumbar spinal stenosis, possibly due to direct hard bony encroachment over time as opposed to softer scar or indirect compression of the neural elements. Investigation into the prognosis and optimal timing of surgery with navigated decompression of HO may be further characterized through additional studies involving the evaluation of a larger patient population, which includes prospectively collected data.

## Conclusions

Recurrent neural compression due to rhBMP-2-induced HO represents a subset of recurrent lumbar spinal stenosis occurring after spinal arthrodesis. Symptomatic patients presenting with this clinical entity may be at risk for poorer clinical outcomes with respect to function and pain; however, further studies are required to gain deeper insights into this. O-arm-based intraoperative image-guided navigation is a useful adjunct in these circumstances and may allow for a safer and more thorough decompression of the heterotopic bone. Long-term results of this type of decompression should be further studied.
